# Antibiofilm Activity of Kefir Probiotic Lactobacilli Against Uropathogenic *Escherichia coli* (UPEC)

**Published:** 2020

**Authors:** Maryam Ghane, Laleh Babaeekhou, Seyedeh Sepideh Ketabi

**Affiliations:** Department of Biology, Islamshahr Branch, Islamic Azad University, Islamshahr, Iran

**Keywords:** Kefir, *Lactobacillus paracasei*, *Lactobacillus rhamnosus*, Probiotics, Urinary tract infection, Uropathogenic *E. coli*

## Abstract

**Background:**

Inhibition of biofilm formation is essential for the prevention and treatment of urinary tract infection. This study was aimed to identify the probiotic potential of Lactobacillus strains isolated from kefir and evaluate their antimicrobial and antibiofilm activities against Uropathogenic *Escherichia coli* (UPEC).

**Methods:**

Twelve Lactobacillus strains were evaluated. Antimicrobial and antibiofilm activities of Cell Free Supernatant (CFS) of the Lactobacillus strains against UPEC isolates were evaluated by agar well diffusion method and crystal violet assay, respectively. Probiotic potential of selected isolates was assessed by analyzing their tolerance to acidic pH and bile salts, auto-aggregation ability, co-aggregation with *Escherichia coli (E. coli)* and hemolytic activity. The isolates were identified by phenotypic and 16S rRNA gene sequencing.

**Results:**

The CFS of all lactobacilli strains was able to inhibit UPEC isolates even after neutralization. Four out of 12 isolates inhibited the biofilm formation by UPEC in the range 62–75%. The viability under acidic condition varied among the isolates ranging from 6–89.8%. All the isolates could tolerate the 0.3% bile and eight isolates showed the adaptation time of less than 1 *hr*. All the strains exhibited co-aggregation with *E. coli*. Auto-aggregation was highly correlated with co-aggregation of all lactobacilli strains with *E. coli* (r=0.889, p<0.001). The isolates with satisfactory probiotic potential and higher ability of biofilm inhibition and antibacterial activity belonged to the species *Lactobacillus rhamnosus* and *Lactobacillus paracasei*.

**Conclusion:**

All four selected probiotic strains exhibited antimicrobial and antibiofilm activities, which suggest potential applications for controlling or preventing infections caused by UPEC.

## Introduction

Urinary Tract Infection (UTI) is one of the most common bacterial infections that annually affect 150 million people worldwide. Among the microorganisms associated with UTI, uropathogenic *Escherichia coli* (UPEC) is the most important cause of community-acquired (90%) and nosocomial UTIs (50%) ^1^. UPEC forms microcolonies known as biofilms on the surface of urethral catheters, as well as on mucosa of urinary bladder ^2^. Biofilm protects bacteria against host immune response and antimicrobial therapy by encapsulating them in an extracellular matrix. In addition, close association of bacteria enables easy transfer of resistance determinants between the bacteria residing in the biofilm ^2^. This will improve drug resistance and makes treatment of UTIs more difficult.

With the emergence and spread of new antibiotic resistant isolates, antibiotic free treatments have gained popularity in recent years. Among these treatment approaches, the use of probiotics is a promising alternative for control of UTIs. It has been stated that some probiotics are able to adhere to uroepithelial cells and inhibit the growth of pathogenic bacteria ^3^. In addition, oral administration of lactobacilli can colonize these microorganisms in urinary tract after intestinal colonization ^3^.

Probiotics are defined as live microorganisms that, when applied in adequate amounts, provide health benefits to their host ^4^. Among the probiotic microorganisms, Lactic Acid Bacteria (LAB) have a long history of safe use in fermented and non-fermented food. Although a considerable number of probiotic microorganisms are commercially available all over the world, screening for new strains is still of great industrial interest.

Kefir is one of the most potent sources of probiotics. It is a popular fermented milk which confers various beneficial health ^5^. Kefir is traditionally made by kefir grains as starter. The kefir grains are composed of polysaccharide and protein matrix which is populated by a diverse group of LAB and yeast ^5^. The beneficial effects of kefir is not only due to the bioactive peptide and soluble polysaccharide (Kefiran), but also its undefined microbial composition and secondary metabolites ^6^. Previous reports described the antimicrobial activity of LAB isolates from kefir against pathogenic bacteria ^7–9^. However, no report has documented the antibiofilm activity of kefir isolates against UPEC. The purpose of this study was to identify and characterize the probiotic potential of LAB isolated from kefir and to investigate the antimicrobial and antibiofilm properties of the isolates against UPEC.

## Materials and Methods

### Microorganisms and growth conditions

For the isolation of LAB strains, 10 *g* of kefir grains from Kefirnoosh Company (Iran) were added to 200 *ml* of sterilized milk and incubated at 21*°C* for 24 *hr*. The grains were then passed through a sterilized strainer and homogenized with 90 *ml* of sterile saline containing 0.9% NaCl and 0.1% bacto peptone (Difco Laboratories). Serial dilutions were made using the homogenized suspensions of kefir grains in sterilized saline and aliquots were spread plated onto the Man, Rogosa and Sharpe (MRS) agar (Merk, Germany). The plates were incubated in anaerobic condition (Gaspak EZ, Difco) at 30*°C* for 48 *hr*. After incubation, the bacterial colonies were picked and streaked on a fresh MRS agar plate. Catalase negative, gram positive and rod shape isolates were identified as lactobacilli and stocked in skim milk with 20% (*v/v*) glycerol (Merk, Germany) at −80*°C*.

For UPEC isolation and cultivation, urine samples from the patients with urinary tract infection referred to Imam Khomeini hospital, Karaj, Iran were cultured on MacConkey agar (Merck, Germany) and incubated at 37*°C* for 24 *hr*. The identification of the isolates was performed using the biochemical tests ^10^. *Escherichia coli (E. coli)* PTCC 1399 was purchased from the Iranian Research Organization for Science and Technology (IROST), grown in Trypticase Soy Broth (TSB) (Merck, Germany), and incubated at 37*°C* under aerobic conditions. All *E. coli* strains were maintained at −80*°C* in the TSB containing 20% (*v/v*) glycerol.

### Antimicrobial susceptibility testing of UPEC isolates

The antimicrobial susceptibility testing was performed by Kirby Bauer's disk diffusion method on Mueller-Hinton agar (Merk, Germany) according to the Clinical and Laboratory Standard Institute (CLSI) guidelines ^11^, using 8 antibiotics including Ampicillin (10 *μg*), Cefotaxime (30 *μg*), Ceftazidime (30 *μg*), Imipenem (10 *μg*), Gentamicin (10 *μg*), Amikacin (30 *μg*), Tetracycline (30 *μg*) and Ciprofloxacin (5 *μg*) (MAST, UK).

### Antimicrobial activity of culture supernatant of LAB strains

Antimicrobial activity of cell free supernatant (CFS) of lactobacilli strains against UPEC isolates was assessed by agar well diffusion method as described previously ^12^. Overnight culture of LAB strains was centrifuged at 9000×*g* for 10 *min* at 4*°C* and CFS was sterilized using syringe filters (0.22 *μm* pore size). Then, 100 *μl* of each UPEC isolates (0.5 McFarland turbidity) was spread onto the Mueller-Hinton agar plate and 6 *mm* wells were cut with a sterilized Pasteur pipette. Next, 100 *μl* of CFS was poured into well and the plates were incubated at 37*°C* for 24 *hr*. The zone of inhibition around the wells was measured in millimeters. To counteract the effect of reduced pH on antimicrobial activity, the pH of CFS was adjusted to 6.5 (NaOH 1N, Merk) and antimicrobial activity was determined as mentioned above. *E. coli* PTCC 1399 was used as the control in the experiments.

### Biofilm formation by UPEC isolates

Biofilm formation was assessed in 96-well micro-titer plates, according to the method described by Ste-panović *et al* ^13^. Briefly, overnight culture of *E. coli* strains was diluted to get 0.5 McFarland turbidity. After that, 1:100 dilution of this suspension was prepared in fresh Luria-Bertani (LB) medium (Merck, Germany) and 100 *μl* of diluted suspension was poured into the well of microtiter plate and the plates were incubated at 37*°C* for 48 *hr*. The attached cells were then fixed by 200 *μl* of methanol (96%) (Merk, Germany) for 15 *min*. Then, 150 *μl* of 2% crystal violet was added to each well and the plates were incubated for 15 *min* at room temperature. Finally, 150 *μl* of 33% acetic acid was added to each well and the OD550 was measured using a microtiter-plate reader (Bio-Rad, USA). The isolates, which had the highest biofilm formation potential, were used for antibiofilm assay.

### Inhibition of biofilm formation

Inhibition of biofilm formation by CFS of LAB strains was performed as described previously by Kaur *et al* with some modification ^14^. Briefly, 100 *μl* of Brain Heart Infusion (BHI) broth (Merck, Germany) was added to each well of a microtiter plate. Then, 100 *μl* of CFS was added to each well except for negative control, which contained MRS broth. At the final step, 20 *μl* of overnight culture of *E. coli* isolates (OD595=0.1) was added to each well and the plates were incubated at 37*°C* for 48 *hr*. The biofilm formation was determined as described above. The percentage of biofilm inhibition was calculated by the following equation:

Percentage inhibition=100–[(OD_595_ of experimental wells ×100)/OD_595_ of negative control well].

### Probiotic potential of lactobacilli

***Acid and bile tolerance of LAB isolates:*** Overnight cultures of LAB strains were centrifuged (Hettich, Germany) at 7500×*g* for 5 *min* at 4*°C*. The pellet was then resuspended in phosphate-saline buffer (10 *mM* Na_2_HPO_4_, 1 *mM* KH_2_PO_4_, 140 *mM* NaCl, 3 *mM* KCl) (PBS pH=6.5) at a concentration of 10^9^ Colony-Forming Units (CFU) *ml*^−1^. Cell suspension was diluted 1×10^−1^ in MRS broth at pH=3 and incubated for 3 *hr* at 30*°C*. Samples were taken at 0 and 3 *hr* and serially diluted in physiological saline solution. The pH tolerance of the isolates was evaluated by counting the viable cells on MRS agar plates after 48 *hr* incubation at 30*°C.* The survival rate of LAB was estimated as follows:

$$\%\;\text{survival}\;=\;\frac{\log\;\text{CFU}\;\text{of}\;\text{viable}\;\text{cells}\;\text{survived}}{\log\;\text{CFU}\;\text{initial}\;\text{viable}\;\text{cells}\;\text{inoculated}}\times 100^{15}$$

The effect of bile salts on the growth rate of LAB strains was evaluated as described previously ^16^. Briefly, overnight cultures of LAB isolates were inoculated (1%) into the MRS broth in the presence (Test) and absence (Control) of 0.3% Oxgall (Sigma-Aldrich, USA). The tubes were then incubated at 37*°C* for 9 *hr* and the Optical Density (OD) at 620 *nm* was measured every hour. Tolerance to bile salts was determined based on the time needed to increase the absorbance at 620 *nm* by 0.3 units in MRS broth with and without 0.3% bile salts. The difference between times (*hr*) to obtain 0.3 units between the culture media was considered as the Adaptation Time (AT).

### Auto-aggregation assay

Overnight culture of bacteria was centrifuged at 5000×*g* for 20 *min* at 4*°C* and the pellet was resuspended in PBS pH=7 (10^8^ *CFU.ml*^−1^). The suspension was incubated for 24 *hr* at 30*°C* and the absorbance was recorded before and after incubation at 600 *nm*. The auto-aggregation percentage was determined as [1−(A_t_/A_0_)×100] where At represents the absorbance at 24 *hr* and A0 absorbance at time 0 ^17^.

### Co-aggregation assay

LAB suspensions were prepared (10^8^ *CFU.ml*^−1^) as described above. A bacterial suspension of *E. coli* (PTCC 1399) was also prepared at the same concentration and mixed with equal volumes (500 *μl*) of the LAB strains. The mixture was then incubated at 37*°C* without shaking and the OD 600 *nm* was recorded after 24 *hr*. The co-aggregation percentage was determined as follows:

[(A_pathog_+A_LAB_)/2-(A_mix_)/(A_pathog_+A_LAB_)/2]×100, where A_pathog_ and A_LAB_ are the OD of tubes containing pathogens or LAB strains respectively, and A_mix_ represents the OD of the mixture ^18^.

### Hemolytic activity

Overnight culture of LAB strains was plated on blood agar plates containing 5% (*v/v*) sheep blood and incubated at 30*°C* for 24–48 *hr*. No hemolysis was scored as negative (−) and alpha or beta hemolysis was recorded based on greenish or clear zone around the isolates, respectively.

### Identification of LAB isolates by 16S rRNA gene sequencing and phenotypic test

The LAB strains were identified by using 16S rRNA gene sequencing. Genomic DNA of the isolates was extracted using SinaPure DNA extraction kit (Sinaclone, Iran) and 16S rRNA gene was amplified by prokaryotic universal primers (27F; 5′-AGAGTTTGA TCCTGGCTCAG-3′ and 1492R; 5′-GGTTACCTTG TTACGACTT-3′ as described previously ^8^. The PCR products were purified and sequencing was performed by Microgene Company (Korea). The sequences were then aligned with blast program (http://www.ncbi.nlm.nih.gov/BLAST/) to determine the closest known phylogenetic relatives of the sequenced gene. The phenotypic tests including growth at different temperatures and carbohydrate fermentation were performed to confirm the identification ^19^.

### Statistical analysis

Statistical analysis was carried out by SPSS software (Version 20). All the data are represented as mean±standard deviation (SD) of three independent experiments. The significant differences between mean values were determined by Tukey's test. Correlation coefficients were calculated between auto-aggregation and co-aggregation, and biofilm inhibition ability and antagonistic activity. p<0.05 was considered significant.

## Results

### Biochemical identification of UPEC and antimicrobial susceptibility testing

All the urine isolates formed typical red colonies on MacConkey agar indicating fermentation of lactose. All the isolates were indole and methyl red positive, Voges-Proskauer, citrate and urea negative, and motile. Accordingly, all were confirmed as *E. coli*. The results of antibiotic susceptibilities of UPEC isolates are shown in table 1. Five out of 12 isolates were multidrug resistant and exhibited resistance to at least one agent in three or more antimicrobial categories. These isolates were used for evaluation of antimicrobial activity of lactobacilli isolates.

**Table 1.** Antibiotic susceptibility pattern of UPEC isolates

**Antibiotics**	**UPEC isolates**											

	**EC1**	**EC2**	**EC3**	**EC4**	**EC5**	**EC6**	**EC7**	**EC8**	**EC9**	**EC10**	**EC11**	**EC12**
**Ampicillin**	R	R	R	S	R	R	S	R	R	S	R	R
**Gentamicin**	S	S	R	R	S	S	S	S	R	R	S	S
**Amikacin**	R	S	S	S	S	R	S	S	S	S	R	R
**Imipenem**	R	S	S	S	S	S	S	S	S	R	R	S
**Tetracycline**	R	R	S	S	R	S	R	S	R	R	R	R
**Cefepime**	S	S	S	S	S	S	S	R	S	S	R	S
**Ceftazidime**	S	S	S	R	S	S	R	S	R	S	R	R
**Ciprofloxacin**	R	S	S	S	S	S	S	S	S	R	S	R

Denotes for Resistant (R), Intermediate (I) and Susceptible (S).

### Antimicrobial activity of CSF of probiotics

In the present study, 12 catalase negative, gram positive and rod shape strains were isolated from kefir grains and their antimicrobial activities were assessed against UPEC isolates. The UPEC isolates showed sensitivity to CFS of all probiotics (Table 2). Seven out of 12 lactobacilli strains showed high antagonistic activity against all UPEC isolates (inhibition zone of 13.6–15.9 *mm*). The CFS of all lactobacilli isolates was also active against the control strain. Among the isolates, strain LAB2 showed the highest antibacterial activity against EC10 (p<0.05). The CFS of all LAB strains also displayed antagonistic activity even after neutralization.

**Table 2.** Antagonistic activity of lactobacilli CFS against UPEC isolates by agar well diffusion method

**Lactobacilli strains**	**Zones of inhibition (*mm*)± S.D**					

	***E. coli* PTCC 1399**	**EC1**	**EC9**	**EC10**	**EC11**	**EC12**
**LAB1**	14.3±1.2^a^	15.1±.07 ^a^	14.45±0.2 ^ab^	14.3±0.2 ^bc^	14.9±0.2 ^a^	15.1±0.2 ^a^
**LAB2**	12.2±1.3^ab^	14.3±0.3 ^bc^	14.7±0.14 ^ab^	15.9±0.14 ^a^	14.5±0.35 ^a^	15.3±0.14 ^a^
**LAB3**	10.3±1.1^bcd^	12.4±0.1^d^	12.7±0.3 ^de^	13.2±0.3^de^	12.9±0.14 ^b^	13.3±0.07^cd^
**LAB4**	12.3±0.6^ab^	14.6±0.1 ^bc^	14.4±0.2 ^ab^	14.7±0.14 ^bc^	15. ±0.2 ^a^	15.3±35 ^a^
**LAB5**	12.7±0.6^ab^	14.3±0.1 ^bc^	14±0.35 ^bc^	14.2±0.07 ^bc^	13.6±0.14 ^b^	14.2±0.07 ^abc^
**LAB6**	8.7±0.6^d^	11.3±0.2^e^	11.4±0.3^f^	11.9±0.2 ^f^	11.2±0.3^c^	11.3±0.07^e^
**LAB7**	12.3±1.1^ab^	14.3±0.7 ^bc^	14±0.3 ^bc^	14.9±0.07^b^	15.2±0.3^a^	14.8±0.07 ^ab^
**LAB8**	10.7±0.6^bcd^	12.3±0.2^d^	12.1±0.3 ^ef^	12.7±0.14^ef^	12.7±0.2^b^	12.8±0.07^d^
**LAB9**	9.3±0.6c^d^	12.3±0.2^d^	12.7±0.14^de^	12±0.2^f^	13.2±0.3 ^b^	13.7±0.21 ^bcd^
**LAB10**	11.7±0.6^bc^	14.4±0.7 ^bc^	14.6±0.2 ^ab^	13.9±0.2^cd^	14.7±0.07 ^a^	14.3±0.85 ^abc^
**LAB11**	14.3±1.1^a^	14.9±0.2 ^ab^	15.4±0.3^a^	15±0.3^b^	15.2±0.2^a^	14.9±0.21 ^ab^
**LAB12**	11.3±0.6^bc^	13.4±0.1^c^	13.25±0.2^cd^	12.7±0.2 ^ef^	13.3±0.14 ^b^	13.3±07^cd^

Data are expressed as mean±standard deviation (n=3). Means within the same column with different superscript letters are statistically different based on Tukey's test (p<0.05).

### Antibiofilm activity of CFS against UPEC

**Figure 1.**
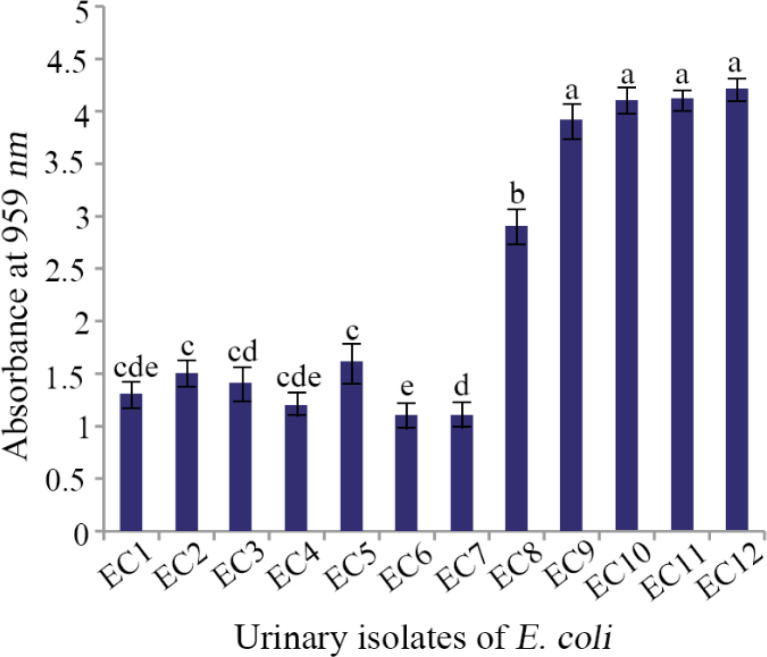
The biofilm formation ability of UPEC isolates. Data are expressed as mean±standard deviation (n=3). Different letters indicate significant differences between *E. coli* isolates based on Tukey's test (p<0.05).

Before determining the inhibitory effect of CFS on biofilm formation by UPEC, the biofilm forming ability of all *E. coli* isolates was tested. Four out of 12 *E. coli* isolates displayed the highest biofilm forming potential (p<0.05) and were used for evaluation of antibiofilm activity of LAB strains (Figure 1).

The CFS of all LAB strains resulted in more than 30% inhibition of biofilm formation by *E. coli* isolates (Table 3). Seven out of 12 strains exhibited more than 50% inhibition against all four urinary isolates and the highest inhibition was observed by LAB7 and LAB4 (more than 70% for both) (p<0.05). Pearson correlation coefficient analysis showed no correlation between biofilm inhibition and antagonistic activity of lactobacilli strains against *E. coli* isolates (r=0.297, p>0.05).

**Table 3.** The inhibitory activity of CFS against biofilm formation by UPEC isolates

**Isolates**	**Inhibition of biofilm formation (%)**			

	**EC9**	**EC10**	**EC11**	**EC12**
**LAB1**	48±2 ^ef^	47±4.4 ^de^	46±3^f^	50. 7±1.5^def^
**LAB2**	62.3±2.5^c^	64.3±1.1 ^b^	65.7±2.5^c^	65±2 ^c^
**LAB3**	32.3±2^g^	31.3±0.6 ^f^	34±1^g^	31.3±3 ^g^
**LAB4**	71.7±1.5 ^ab^	73±1 ^a^	72.7±2.5 ^ab^	73. 7±1.5 ^ab^
**LAB5**	55.3±2.3^e^	55±2.6 ^c^	56.3±1.5^d^	56±2.6 ^d^
**LAB6**	50±2^de^	50±1.5 ^cd^	52.3±0.6 ^de^	51±3.6 ^def^
**LAB7**	73.7±1.5 ^a^	73.3±0.6 ^a^	73. 7±1.5 ^a^	75. 7±3.2^a^
**LAB8**	43±2^f^	43.3±1.1 ^e^	44±2^f^	43.3±2.1 ^f^
**LAB9**	47.7±1.5 ^ef^	48±1 ^de^	44. 7±1.5^f^	48.3±2.5 ^def^
**LAB10**	67.3±1.5^bc^	69.7 a	66. 7±1.5 ^bc^	67.3±3.5 ^bc^
**LAB11**	48.7±3^ef^	50±1 ^cd^	46.3±2.5 ^ef^	47.3±2.1^ef^
**LAB12**	51.3±1.5 ^de^	54±1 ^c^	52.3±3 ^de^	54±2.6 ^de^

Data are expressed as mean±standard deviation (n=3). Means within the same column with different superscript letters are statistically different based on Tukey's test (p<0.05).

### Probiotic properties of lactobacilli

***Acid and bile tolerance:*** Acid and bile tolerance of the isolates are shown in table 4. For two strains (LAB2 and LAB10) survival at pH=3 was significantly higher (p<0.05). LAB4, LAB7 and LAB12 showed the moderate acid tolerance and the lowest acid tolerance was observed for LAB3 and LAB11 (p<0.05) after 3 *hr* of exposure to pH=3 (Table 4).

**Table 4.** Acid and bile salts tolerance of LAB strains

**Isolates**	**Acid tolerance (survival %)**	**Bile tolerance (Time needed for increasing 0.3 units)**		

		**MRS (h)**	**MRS+0.3% bile (h)**	**Adaptation time (h)**
**LAB1**	21.52±6.5^def^	2.93±0.09	3.74±0.1	.79±0.16^b^
**LAB2**	89.79±4^a^	4.04±0.05	4.5±0.04	.42±0.06^bcd^
**LAB3**	7.31±1.45^ef^	4.5±0.04	6.36±0.12	2.05±0.15^a^
**LAB4**	48.41±8.1^bc^	3.79±0.14	4.06±0.05	.36±0.05^bcd^
**LAB5**	22.4±7.27^def^	3.55±0.06	5.57±0.09	2.02±0.16^a^
**LAB6**	24.42±3.2 ^def^	4.51±0.17	6.68±0.03	2.18±0.17^a^
**LAB7**	52.51±8.1^b^	3.64±0.02	4.02±0.03	.37±0.05^bcd^
**LAB8**	44.11±4.5^ef^	4.67±0.02	6.68±0.04	2.00±0.2^a^
**LAB9**	28.4±2.54^cde^	4.04±0.03	4.67±0.02	.62±0.04^b^
**LAB10**	80.07±12.63^a^	3.69±0.04	4.15±0.04	.46±0.1^bc^
**LAB11**	5.89±1.35^f^	3.9±0.07	4.66±0.04	.75±0.11^b^
**LAB12**	32.77±2.37^bcd^	2.95±0.05	3.57±0.09	.62±0.13^b^

Data are expressed as mean±standard deviation (n=3). Means within the same column with different superscript letters are statistically different based on Tukey's test (p<0.05).

All the isolates could tolerate the 0.3% bile for 3 *hr*. Also, 8 out of 12 isolates showed the shortest adaptation time (p<0.05) of less than 1 *hr* (Table 4).

### Auto-aggregation, co-aggregation and safety assessment

**Figure 2.**
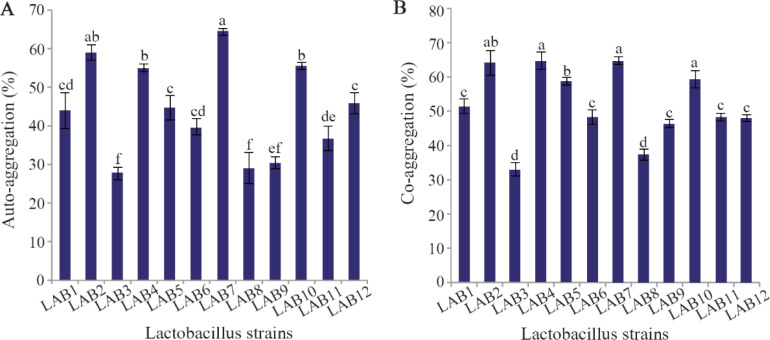
Auto-aggregation (A) and co-aggregation of LAB strains with *E. coli* (B). Data are expressed as mean±standard deviation (n=3). Different letters indicate significant differences between isolates based on Tukey's test (p<0.05).

The auto-aggregation and co-aggregation of the LAB strains are presented in figure 2. Strains LAB2 and LAB7 showed the highest auto-aggregation (58.9 and 64.42%, respectively, p<0.05). All LAB strains exhibited co-aggregation with *E. coli*. The co-aggregation values of LAB strains varied between 33–64.7%. Four out of 12 isolates showed the highest co-aggregation values (p<0.05). To examine whether the autoaggregation and co-aggregation abilities of the strains with *E. coli* were related phenotypes, the Pearson correlation coefficient (r) was conducted. Statistically significant correlation was observed between auto-aggregation and co-aggregation of all lactobacilli strains with *E. coli* (r=0.889, p<0.001). Hemolytic activity was not detected in any LAB strains.

### Identification of the isolates

**Figure 3.**
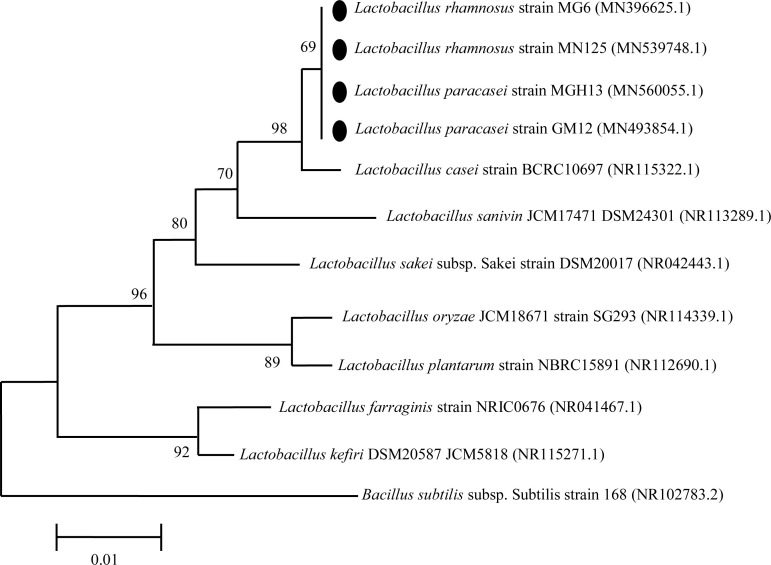
Phylogenetic relationship of selected strains among members of the genus Lactobacillus in the neighbor joining phylogenetic tree. The tree was generated on the basis of 16S rDNA sequences. Accession numbers are in parentheses. *Bacillus subtilis* was used as out groups.

Based on acid and bile tolerance, auto-aggregation and co-aggregation properties and antimicrobial and antibiofilm activities, four isolates (LAB2, LAB4, LAB7 and LAB10) were selected for phenotypic and molecular identification. The 16S rDNA of LAB2, LAB4, LAB7 and LAB10 isolates was sequenced, and the isolates were identified by alignment of 16S rDNA sequences as *Lactobacillus paracasei (L. paracasei)* strain GM12 (Genbank accession no. MN493854), *L. paracasei* strain MGH13 (Genbank accession no. MN560055), *Lactobacillus rhamnosus (L. rhamnosus)* strain MG6 (Genbank accession no. N396625) and *Lactobacillus rhamnosus (L. rhamnosus)* strain MN125 (Genbank accession no. MN539748), respectively. Strains identified as *L. rhamnosus* were able to grow at 45*°C* and ferment mannitol, sorbitol, ribose and sucrose, but not xylose. In comparison, isolates identified as *L. paracasei* were not able to ferment any of the tested carbohydrates and grow at 45*°C*. The results of phenotypic testing confirmed the molecular identification by 16S rRNA gene sequencing. The phylogenetic relationship of selected Lactobacillus strains among the members of the genus Lactobacillus is shown in figure 3.

## Discussion

Nowadays, inhibition of biofilm formation by pathogenic bacteria has become an attractive therapeutic target ^20^. UPEC is well known for its ability to form strong biofilm on the surface of urethral catheter and bladder epithelial cells ^2^. The results obtained in the present study also confirmed the biofilm forming abilities of UPEC isolates in 96-well microtiter plates. Biofilm formation plays a significant role in uropathogenicity ^21^. In addition, biofilms are resistant to the action of antimicrobial drugs as most of the available antimicrobial agents are active only against the planktonic forms of bacteria. Therefore, probiotic strains that have both anti-biofilm and antimicrobial activities against UPEC can be of clinical importance.

In this study, 12 lactobacilli strains were screened for their antimicrobial potential against UPEC isolates, out of which seven isolates showed high antagonistic activity against all UPEC isolates. Different species of lactobacilli are known to produce compounds with antimicrobial properties, including low molecular weight compounds, antimicrobial peptides (Bacteriocins) and organic acids ^22^. It seems that the antimicrobial activity of the lactobacilli strains isolated in this study was not due to the overproduction of acid, since antimicrobial activity was evident even after neutralizing CFS. It can be concluded that other molecules may also be involved in their inhibitory activity against UPEC isolates.

The antibiofilm activity of lactobacilli strains against pathogenic bacteria has been investigated previously. It has been reported that CFS of fecal lactobacilli isolates was able to prevent the *Vibrio cholerae* biofilm formation by more than 90% ^14^. Similarly, the antibiofilm activity of *Lactobacillus plantarum (L. plantarum)* and *Lactobacillus pentosus (L. pentosus)* CFS against *Pseudomonas aeruginosa* and *Klebsiella pneumoniae* has been documented ^23^. In the present study, the LAB isolates from kefir grains were screened for their biofilm inhibition potential against uropathogenic *E. coli*. The antibiofilm activity of lactobacilli strains against ciprofloxacin resistant UPEC has been discussed previously ^24^. However, no documented report concerning the inhibitory activity of LAB isolates from kefir grains against UPEC isolates is available. In the present study, the CFS of all kefir isolates caused reduction in the biofilm formation of all uropathogenic strains. However, biofilm inhibition ability varied significantly among lactobacilli strains (p<0.05). Among the lactobacilli strains, *L. rhamnosus* MG6 and *L. paracasei* MGH13 showed the highest biofilm inhibition potential. This is the first study concerning the antibiofilm activity of kefir isolates against UPEC isolates. It has been documented that lactobacilli CFS contains a variety of bioactive compounds including biosurfactant and exopolysaccharides, which can prevent biofilm formation of pathogenic bacteria ^25^. The amount of biosurfactant or exopolysaccharide produced by each isolate, which depends on its genetic potency, can affect the amount of its antibiofilm activity. The antibiofilm activity of CFS may also be due to its antimicrobial activity, which inhibits bacterial growth and biofilm formation. However, in the present study, no correlation between biofilm inhibition and antagonistic activity against *E. coli* isolates was observed (r=0.279, p>0.05).

In this study, lactobacilli strains were screened for their probiotic properties such as acid and bile tolerance, two key features that enable them to survive and grow in the gastrointestinal conditions ^26^. The acidic conditions of the stomach are the primary defense mechanism that probiotics must overcome. In the present study, most of the lactobacilli strains showed resistance to low pH. The ability of lactobacilli to survive at low pH was also reported in the previous studies ^15,26^. In our study, the survival ability of the lactobacilli strains varied significantly (p<0.05). In this regard, Tokatl *et al* documented the strain specific survival of lactobacilli originated from traditional pickles ^26^.

Bile resistance is an important feature for the selection of bacteria, since gut contains high concentrations of bile salts, which are toxic for bacteria ^27^. In our study, all of the selected isolates were able to grow in 0.3% of bile salts, similar to the concentration found in the small intestine ^8^. However, the time required for growing the isolates in culture media with and without bile salts varied between the strains. Our results are in agreement with the previous study, which demonstrated that lactobacilli strains possessed high resistance to 0.3% of bile salts ^28^.

Colonization of probiotic bacteria in the gut is considered as a desirable feature ^29^. In this regard, adhesion to the intestinal epithelial cells, which is an essential step for colonization, is an important factor in selecting a strain as probiotic ^26^. Auto-aggregation of probiotic bacteria is used as a measurement directly related to adhesion capacity of probiotic bacteria to cell monolayers ^30^. In the present study, significant differences (p<0.05) in auto-aggregation values were observed between lactobacilli strains. Among the lactobacilli isolates, *L. rhamnosus* MG6 and *L. paracasei* GM12 showed the highest auto-aggregation activity (p<0.05). Ramos *et al* also demonstrated variation in auto-aggregation among lactobacilli strains ^31^. Autoaggregation is a phenomenon that has previously been shown to be dependent on production of exopolysaccharide (EPS). The amount of EPS produced by each isolate reflects the rate of auto-aggregation ^32^.

Co-aggregation between lactobacilli and pathogenic bacteria provides a barrier that prevents their adhesion to the urinary and intestinal epithelial cells. All the lactobacilli isolated in this study exhibited interaction with *E. coli*. However, co-aggregation percentages varied significantly (p<0.05). Among the lactobacilli isolates, four strains (LAB2, LAB4, LAB10 and LAB7), identified as strains of *L. paracasei* and *L. rhamnosus*, showed high percentages of co-aggregation with *E. coli* in the range of 58–68%. In a study conducted by Tareb *et al*, the co-aggregation percentage of *L. rhamnosus* CNCM-I-3698 and *L. farciminis* CNCM-I-3699 with *E. coli* was found to be 38.2 and 34.5, respectively ^33^. Comparing to their results, *L. rhamnosus* strains isolated in this study showed the higher percentages of coaggregation potential. The co-aggregation potential of *L. rhamnosus* and *L. paracasei* strains isolated in this study may be an effective host defense mechanism against infections caused by *E. coli*. In addition, the coaggregation phenomenon can cause the probiotic to be in close contact with the pathogenic bacteria and thus increase the efficiency of the antimicrobial compounds produced by the probiotics.

In the present study, strains with strong autoaggregation ability were also well co-aggregated with *E. coli*. The correlation coefficients between autoaggregation and co-aggregation showed that autoaggregation was highly correlated with co-aggregation of all lactobacilli strains with *E. coli* (r=0.889, p<0.01). Our findings suggest that auto-aggregation ability is related to co-aggregation property of each lactobacilli isolates.

In this study, four Lactobacillus strains, including two *L. paracasei* strains (LAB2 and LAB4) and two *L. rhamnosus* strains (LAB7, LAB10) were selected and identified by molecular methods. The phylogenetic tree obtained in this study, showed the close relationship between our isolates and *Lactobacillus casei (L. casei)*. It has been stated that *L. paracasei*, *L. rhamnosus* and *L. casei* are phenotypically and phylogenetically closely related; and are regarded as the *L. casei* group. Members of this group are widely used in fermented dairy products and food supplements ^34^.

## Conclusion

In the present study, four lactobacilli strains with satisfactory probiotic potential were isolated from kefir grains. The isolates exhibited high auto-aggregation and co-aggregation activities and were able to inhibit biofilm formation by uropathogenic *E. coli*. Our findings suggest that Lactobacillus strains isolated in this study may be promising probiotics for prevention and treatment of UTIs. However, *in vivo* studies are necessary for future applications of these microorganisms as probiotics.
